# Dietary habits and *Helicobacter pylori* infection: a cross sectional study at a Lebanese hospital

**DOI:** 10.1186/s12876-018-0775-1

**Published:** 2018-04-16

**Authors:** Shafika Assaad, Rawan Chaaban, Fida Tannous, Christy Costanian

**Affiliations:** 1 Faculty of Sciences, Lebanese University, Beirut, Lebanon; 2 International Committee of the Red Cross, Beirut, Lebanon; 3 Faculty of Sciences, Beirut Arab University, Beirut, Lebanon; 4 School of Kinesiology and Health Science, York University, Toronto, ON M3J1P3 Canada

**Keywords:** Dietary habits, *Helicobacter pylori*, Socio-demographic factors, Lebanon

## Abstract

**Background:**

To examine the association between dietary habits and *Helicobacter pylori* (*H. pylori*) infection among patients at a tertiary healthcare center in Lebanon.

**Methods:**

This cross-sectional study was conducted on 294 patients in 2016, at a hospital in Northern Lebanon**.** Participants were interviewed using a structured questionnaire to collect information on socio-demographic and lifestyle characteristics; dietary habits were ascertained via a short food frequency questionnaire (FFQ). *H. pylori* status (positive vs. negative) was determined after upper GI endoscopy where gastric biopsy specimens from the antrum, body, and fundus region were collected and then sent for pathology analysis. Multivariable logistic regression was conducted to identify the association between socio-demographic, lifestyle, dietary and other health-related variables with H pylori infection.

**Results:**

The prevalence of *H. pylori* infection was found to be 52.4% in this sample. Results of the multivariable analysis showed that *H. pylori* infection risk was higher among participants with a university education or above (OR = 2.74; CI = 1.17–6.44), those with a history of peptic ulcers (OR = 3.80; CI = 1.80–8.01), gastric adenocarcinoma (OR = 3.99; CI = 1.35–11.83) and vitamin D level below normal (OR = 29.14; CI = 11.77–72.13). In contrast, hyperglycemia was protective against *H. pylori* (OR = 0.18; CI = 0.03–0.89). No relationship between dietary habits and *H. pylori* infection was found in the adjusted analysis.

**Conclusions:**

Socio-demographic and clinical variables are found to be associated with *H. pylori,* but not with dietary factors. Further studies are needed to investigate the effect of diet on *H. pylori* risk.

## Background

*Helicobacter pylori* (*H. pylori*) infection is one of the most prevalent chronic gastric infections, affecting more than 50% of the world population [1–3]^]^. The microorganism is the first formally recognized bacterial carcinogen, leading to the development of various upper gastro-intestinal disorders including gastritis, gastro-duodenal ulcer diseases and gastric cancer [4]. The latter was established to be the second leading cause of cancer-related death worldwide [5, 6]. Epidemiological studies have demonstrated that *H. pylori* infection is most prevalent in developing countries and among populations with low socioeconomic background [2, 7, 8]. In addition to income and education level, living standards such as sanitation and hygiene, crowding index, and source of drinking water have been shown to be risk factors of *H. pylori* [3, 8, 9]. Major variations in prevalence rates were observed among different ethnic groups, suggesting a possible genetic susceptibility [2, 3, 8–10]. Lifestyle factors are also believed to contribute to *H. pylori* infection development. Studies on the association of smoking and alcohol consumption with the infection show conflicting results. While some have found that smoking was associated with an increased risk for *H. pylori,* and that alcohol consumption had no effect on it [11–13], others have concluded that both smoking and alcohol consumption had a protective effect against the infection [14, 15].

Previous studies worldwide have investigated the relationship between dietary patterns and *H. pylori*, with many being published over 20 years ago. Some studies have found that salty, pickled, fermented, or smoked foods increased the risk of *H. pylori* infection [16–18], while another found no association between *H. pylori* and pickled food [19]. Also, high intake of fruits, vegetables or of antioxidants were found to be protective factors infection in some studies [17, 19]. Moreover, Eslami et al. [20] reported that lower consumption of raw vegetables was significantly associated with higher risk of *H. pylori* infection in a group of Iranian students. A recent case-control study of patients with peptic ulcer (*n* = 190) and control group (*n* = 125) in Pune, India, found that meat consumption (OR = 2.35, 95% CI = 1.30–4.23) as well as the consumption of restaurant food increased the risk for *H. pylori* infection, while chili peppers intake was protective against it (OR = 0.20, 95% CI = 0.10–0.37) [11].

Research has also been conducted in the Middle East and North Africa (MENA) region on the association between *H. pylori* and diet [21, 22], however no conclusive evidence on this relationship exists yet. Studies on the prevalence of *H. pylori* infection in Lebanon are scarce [23–25]. In addition, risk factors for *H. pylori* infection, especially lifestyle and dietary factors, have not been comprehensively investigated in in this context. Given the high prevalence of modifiable cardio-metabolic risk factors in the MENA region, and Lebanon in particular, and given the high burden of this infection in developing countries, a study investigating the role of dietary habits in *H. pylori* infection is warranted. This study aims to examine the association between dietary habits and *H. pylori* infection among adult patients undergoing endoscopic examination at a tertiary health care center in Lebanon.

## Methods

### Study design and participants

This cross sectional study was conducted between March 2016 and December 2016, at Centre Hospitalier du Nord- Zgharta, a major tertiary care hospital in the North region of Lebanon. Study participants aged 18 years or above were recruited at the gastroenterological unit as they were being referred for endoscopic examination of the upper gastrointestinal (GI) tract (gastroscopy) to obtain a biopsy**.** A retrospective chart review was conducted to determine study eligibility. Only patients with gastrointestinal (dyspeptic) symptoms, mainly epigastric pain and gastritis and who were undergoing gastroscopy were included in the study. Patients were excluded if they had a history of *H. pylori* eradication therapy, a history of antibiotic, antacid, H_2_ blocker, proton pump inhibitors (PPI), bismuth compound, or nonsteroidal anti-inflammatory drug (NSAID) use during the previous 4 weeks, or had a previous diagnosis of other inflammatory diseases, such as coeliac disease, inflammatory bowel disease or allergies, or had gastric perforation or hemorrhage, or a history of abdominal surgery. Based on these eligibility criteria, a total of 294 participants were consecutively recruited for this study. Informed consent of the participants was obtained and all completed questionnaires were anonymous and confidential. Approval to conduct the present study was granted by the administration of the participating hospital. This study protocol was reviewed and approved by the institutional review board at the Lebanese University.

### Data collection and measures

A structured questionnaire was administered by one trained interviewer during face-to-face interviews prior to the endoscopic examination. The questionnaire was composed of five sections: socio-demographic characteristics that included age, sex, educational level, marital status, occupation and monthly income; lifestyle characteristics gathering information on cigarette and arguileh smoking statuses and frequencies, alcohol consumption, stress, total number of hours slept and frequency and intensity of physical activity; dietary habits and a short food frequency questionnaire (FFQ). The FFQ was adapted from a validated questionnaire used by Yassibas et al. [26], and assessed the frequency of consuming 13 types of food (milk, yogurt, salty cheese, red meat, salami or ham, sausages, hot dogs, hamburgers, chicken, fish, green vegetables, tuberous vegetables, and grains). Frequency was assessed through selecting one of five categories (“less than once per month or none”, “1-2 times a month”, “1–2 times a week”, “3-4 times a week” and “every day”); the last three frequency categories were combined into “once or more per week” for the statistical analysis. Questions on dietary habits were also adapted such as type of drinking water during childhood, coffee consumption, chili pepper consumption, eating rate, food temperature, salt status of dishes, and consumption of food from outside the house.

A clinical section also collected information on history of digestive diseases including gastroenteritis, peptic ulcer, esophagitis and hepatitis; and gastric cancer types and stages. Family history of cancer was also reported. Medical records of patients were also retrieved and reviewed. Information was obtained on biochemical measurements including glycemia level (above normal> 1.2 g/L), HDL (below normal< 0.45 g/L), LDL (above normal> 1.6 g/L), total cholesterol (above normal > 2.1 g/L), triglyceride (above normal> 1.5 g/L), iron level (below normal < 50 μg/L) and vitamin D level (below normal< 20 nanog/L) via medical chart abstraction. These variables were re-coded as binary (normal level versus not) based on widely known cut off levels for each parameter. Systolic and diastolic blood pressures and anthropometrics including height and weight were also collected from patients’ records. Hypertension was defined as having a systolic blood pressure above 140 mmHg or a diastolic pressure above 90 mmHg. Body mass index was calculated by dividing the weight (kilograms) by the square of height (meters) and was classified into four categories: underweight (< 18.5), normal weight (18.5–24.9), overweight (25.0–29.9) and obese (≥30.0).

### Outcome assessment

Identification of the microorganism was done according to standard procedures.

*H. pylori* status (positive vs. negative) was determined after upper GI endoscopy where gastric biopsy specimens from the antrum, body, and fundus region were collected in a plate containing formalin buffer. These samples were then sent to the pathology laboratory and examined by a pathologist. Contamination detection was performed with hematoxylin and eosin (H&E) [27]. Semi-quantitative method of scoring according to the Updated Sydney Classification System was undertaken.

### Statistical analysis

All eligible questionnaires were coded. Student’s *t* -tests was conducted to examine differences in continuous variables including age, duration of smoking, duration of drinking alcohol, and number of coffee cups consumed per day between cases and non-cases. Chi-square analyses were used to compare frequency distributions of categorical variables with the two groups of *H. pylori* infection. Univariate logistic regression was performed to evaluate the crude association between dietary factors and *H. pylori* status. Next, multivariable backward regression analysis was employed to examine the association of risk factors controlling for potential confounders *H. pylori* infection was the dependent variable in all regression models. Odds ratio (OR) and 95% confidence interval (CI) were calculated. Sample size calculations were performed assuming the following parameters: alpha error = 0.05, power = 80%, expected effect size: odds ratio (OR) = 1.4, proportion of people with the outcome (*H. pylori*) = 0.50, thereby yielding a required sample size of at least 200 participants. A two tailed *p*-value of <.05 was considered as statistically significant. All statistical analyses were performed using the Statistical Package for Social Sciences (version 22.0, SPSS, Inc).

## Results

The total number of study participants was 294. The mean age of the sample was 40.55 years (SD ± 14.11), with a proportion of females larger than males (63.3% vs 36.7%). The prevalence of *H. pylori* infection was found to be 52.4% in this sample. Tables 1, 2, 3, 4, 5, and 6 show the differences in terms of characteristics between *H. pylori* positive and *H. pylori* negative subjects *H. pylori* infection was significantly lower among hyperglycemic subjects (*p* = 0.006) and those with vitamin D levels below normal (*p* < 0.001) (Table 2). Lifestyle and dietary factors were similar between *H. pylori* positive and *H. pylori* negative subjects, except for frequency of milk consumption with *H. pylori* being more prevalent among subjects who consumed milk 1–2 times per month and once or more per week in comparison to those who consumed milk less than once per month (*p* = 0.030) (Tables 3 and 4). Table 5 shows that *H. pylori* was more prevalent among subjects with peptic ulcer (p < 0.001); subjects with history of hepatitis C were less likely to be *H. pylori* positive (*p* = 0.022). *H. pylori* infection was more common among subjects with gastric adenocarcinoma (*p* = 0.005) (Table 6). Table 6 highlights results of the bivariate and multivariable logistic analyses. Hyperglycemia (OR = 0.26; CI = 0.08–0.83), vitamin D deficiency (OR = 24.57; CI = 10.78–56.03), consuming milk 1–2 times per month (OR = 2.23; CI = 1.21–4.10), history of peptic ulcer or gastric (OR = 4.20; CI = 2.23–7.90; OR = 3.58; CI = 1.40 = 9.15, respectively), and a history of hepatitis C (OR = 0.19; CI = 0.04 = 0.92) were associated with *H. pylori* infection at the bivariate level. After adjustment for significant variables at the univariate levels and potential predictors as indicated by the literature, the risk of *H. pylori* infection was significantly higher among participants with a university education or above (OR = 2.74; CI = 1.17–6.44) versus those with a lower education level. Patients who reported a vitamin D deficiency were more likely to be *H. pylori* positive than those with normal vitamin D levels (OR = 29.14; CI = 11.77–72.13). Subjects with a history of peptic ulcers were almost 4 times more likely to be *H. pylori* positive (OR = 3.80; CI = 1.80–8.01). Patients with gastric adenocarcinoma (OR = 3.99; CI = 1.35–11.83) were also at a 4 times increased odds of reporting *H. pylori* infection. In contrast, subjects with hyperglycemia were more than 5 times less likely to be *H. pylori* positive (OR = 0.18; CI = 0.03–0.89).

**Table 1:** Percent distribution of socio-demographic characteristics of participants

	Overall *n* = 294	*H. pylori* (−) *n* = 140	*H. pylori* (+) *n* = 154	*p*-value
Age (mean ± SD, years)	40.55 ± 14.11	41.04 ± 14.37	40.10 ± 13.90	0.570
Sex				
Males	108 (36.7)	48 (44.4)	60 (55.6)	0.358
Females	186 (63.3)	93 (50.0)	93 (50.0)	
Marital status				
Non married	90 (30.6)	42 (46.7)	48 (53.3)	0.768
Married	204 (69.4)	99 (48.5)	105 (51.5)	
Education				
Middle	55 (18.7)	31 (56.4)	24 (43.6)	0.379
Secondary	73 (24.8)	33 (45.2)	40 (54.8)	
University and higher	166 (56.5)	77 (46.4)	89 (53.6)	
Income (per month)				
< 660 USD	121 (41.2)	54 (44.6)	67 (55.4)	0.339
≥ 660 USD	173 (58.8)	87 (50.3)	86 (49.7)	
Employment status				
Unemployed	110 (37.4)	49 (44.5)	61 (55.5)	0.365
Employed	184 (62.6)	92 (50.0)	92 (50.0)

**Table 2:** Percent distribution of medical conditions among participants

	Overall *n* = 294	*H. pylori* (−) *n* = 140	*H. pylori* (+) *n* = 154	*p*-value
Body Mass Index (kg/m^2^)				0.083
Underweight (< 18.5)	12 (4.1)	7 (58.3)	5 (41.7)	
Normal weight (18.5–24.9)	145 (49.3)	79 (54.5)	66 (45.5)	
Overweight (25.0–29.9)	78 (26.5)	33 (42.3)	45 (57.7)	
Obese (≥ 30)	59 (20.1)	22 (37.3)	37 (62.7)	
Hypertension				0.527
No	162 (55.1)	75 (46.3)	87 (53.7)	
Yes	132 (44.9)	66 (50.0)	66 (50.0)	
Glycemia				**0.006**
Normal (≤1.2 g/L)	278 (94.6)	128 (46.0)	150 (54.0)	
Above normal (> 1.2 g/L)	16 (5.4)	13 (81.3)	3 (18.8)	
Total Cholesterol				0.092
Normal (≤2.1 g/L)	239 (81.3)	109 (45.6)	130 (54.4)	
Above normal (> 2.1 g/L)	55 (18.7)	32 (58.2)	23 (41.8)	
Triglycerides				0.103
Normal (≤1.5 g/L)	183 (62.2)	81 (44.3)	102 (55.7)	
Above normal (> 1.5 g/L)	111 (37.8)	60 (54.1)	51 (45.9)	
HDL				0.789
Normal (≥0.45 g/L)	140 (47.6)	66 (47.1)	74 (52.9)	
Below normal (< 0.45 g/L)	154 (52.4)	75 (48.7)	79 (51.3)	
LDL				0.971
Normal (≤1.6 g/L)	265 (90.1)	127 (47.9)	138 (52.1)	
Above normal (> 1.6 g/L)	29 (9.9)	14 (48.3)	15 (51.7)	
Iron level				0.830
Normal (≥50 μg/L)	233 (79.3)	111 (47.6)	122 (52.4)	
Below normal (< 50 μg/L)	61 (20.7)	30 (49.2)	31 (50.8)	
Vitamin D level				**< 0.001**
Normal (≥20 nanog/L)	201 (68.4)	134 (66.7)	67 (33.3)	
Below normal (< 20 nanog/L)	93 (31.6)	7 (7.5)	86 (92.5)	
Family history of cancer				
No	151 (51.4)	71 (50.4)	70 (49.6)	0.740
Yes	143 (48.6)	80 (52.3)	73 (47.7)	

Bolded data are significant

**Table 3:** Percent distribution of lifestyle characteristics of participants

	Overall *n* = 294	*H. pylori* (−) *n* = 140	*H. pylori* (+) *n* = 154	*p*-value
Cigarette smoking				
Non Smoker	213 (72.4)	109 (51.2)	104 (48.8)	0.074
Current Smoker	81 (27.6)	32 (39.5)	49 (60.5)	
1–10 cigarettes/day	27 (9.2)	15 (55.6)	12 (44.4)	0.094
11–20 cigarettes/day	37 (12.6)	12 (32.4)	25 (67.6)	
> 20 cigarettes/day	18 (6.1)	5 (27.8)	13 (72.2)	
Duration of smoking (years)	15.74 ± 10.15	15.91 ± 10.14	15.63 ± 10.26	0.904
Waterpipe smoking				
Non Smoker	226 (76.9)	108 (47.8)	118 (52.2)	0.915
Current Smoker	68 (23.1)	33 (48.5)	35 (51.5)	
Daily	28 (9.5)	12 (42.9)	16 (57.1)	0.281
Weekly or monthly	15 (5.1)	10 (66.7)	5 (33.3)	
Occasionally	25 (8.5)	11 (44.0)	14 (56.0)	
Alcohol consumption				
Non drinker	192 (65.3)	92 (47.9)	100 (52.1)	0.984
Current drinker	102 (34.7)	49 (48.0)	53 (52.0)	
One to several times a week	19 (6.5)	12 (63.2)	7 (36.8)	0.144
One to several times a month	83 (28.2)	37 (44.6)	46 (55.4)	
Duration of drinking	11.94 ± 8.04	11.55 ± 7.37	12.30 ± 8.68	0.650
Feeling tense or stressed out				
Not at all	46 (15.6)	21 (45.7)	25 (54.3)	0.455
Occasionally	73 (24.8)	30 (41.1)	43 (58.9)	
A lot of times	57 (19.4)	31 (54.4)	26 (45.6)	
Most of the time	118 (40.1)	59 (50.0)	59 (50.0)	
Sleep per night				
< 7 h per day	136 (46.3)	63 (46.3)	73 (53.7)	0.602
≥ 7 h per day	158 (53.7)	78 (49.4)	80 (50.6)	
Physical activity				
< once per week	227 (77.2)	109 (48.0)	118 (52.0)	0.924
Once per week	31 (10.5)	14 (45.2)	17 (54.8)	
≥ twice per week	36 (12.2)	18 (50.0)	18 (50.0)	
Physical activity intensity				
None	21 (7.1)	11 (52.4)	10 (47.6)	0.090
Light	151 (51.4)	62 (41.1)	89 (58.9)	
Moderate	94 (32.0)	54 (57.4)	40 (42.6)	
Vigorous	28 (9.5)	14 (50.0)	14 (50.0)

**Table 4:** Percent distribution of dietary factors of participants

	Overall *n* = 294	*H. pylori* (−) *n* = 140	*H. pylori* (+) *n* = 154	*p*-value
Drinking water during childhood				
Tap water	123 (41.8)	60 (48.8)	63 (51.2)	0.917
Well water	40 (13.6)	18 (45.0)	22 (55.0)	
Mineral or filtered	129 (43.9)	62 (48.1)	67 (51.9)	
Coffee consumption				
No	80 (27.2)	36 (45.0)	44 (55.0)	0.535
Yes	214 (72.8)	105 (49.1)	109 (50.9)	
Coffee cups/day	5.48 ± 3.97	5.59 ± 4.09	5.37 ± 3.87	0.682
Chilli pepper consumption				
No	140 (47.6)	60 (42.9)	80 (57.1)	0.095
Yes	154 (52.4)	81 (52.6)	73 (47.4)	
Eating rate				
Very fast	83 (28.2)	45 (54.2)	38 (45.8)	0.527
Fast	68 (23.1)	29 (42.6)	39 (57.4)	
Normal	102 (34.7)	47 (46.1)	55 (53.9)	
Slow	41 (13.9)	20 (48.8)	21 (51.2)	
Food temperature				
Cooling/warm	141 (48.0)	63 (44.7)	78 (55.3)	0.540
Hot	80 (27.2)	40 (50.0)	40 (50.0)	
Very hot	73 (24.8)	38 (52.1)	35 (47.9)	
Salt status of dishes				
Very salty	41 (13.9)	18 (43.9)	23 (56.1)	0.478
Salty	126 (42.9)	64 (50.8)	62 (49.2)	
Less salty	66 (22.4)	27 (40.9)	39 (59.1)	
Salt free	61 (20.7)	32 (52.5)	29 (47.5)	
Frequency of drinking milk				
None/Less than once per month	167 (56.8)	90 (53.9)	77 (46.1)	**0.030**
1–2 times per month	61 (20.7)	21 (34.4)	40 (65.6)	
Once or more per week	66 (22.4)	30 (45.5)	36 (54.5)	
Frequency of eating yogurt				
None/Less than once per month	36 (12.2)	12 (33.3)	24 (66.7)	0.062
1–2 times per month	167 (56.8)	89 (53.3)	78 (46.7)	
Once or more per week	91 (31.0)	40 (44.0)	51 (56.0)	
Frequency of eating salty cheese				
None/Less than once per month	31 (10.5)	13 (41.9)	18 (58.1)	0.251
1–2 times per month	75 (25.5)	42 (56.0)	33 (44.0)	
Once or more per week	188 (63.9)	86 (45.7)	102 (54.3)	
Frequency of eating red meat				
None/Less than once per month	30 (10.2)	17 (56.7)	13 (43.3)	0.140
1–2 times per month	72 (24.5)	40 (55.6)	32 (44.4)	
Once or more per week	192 (65.3)	84 (43.8)	108 (56.3)	
Frequency of eating ham				
None/Less than once per month	176 (59.9)	83 (47.2)	93 (52.8)	0.774
1–2 times per month	76 (25.9)	39 (51.3)	37 (48.7)	
Once or more per week	42 (14.3)	19 (45.2)	23 (54.8)	
Frequency of eating sausages				
None/Less than once per month	188 (63.9)	85 (45.2)	103 (54.8)	0.426
1–2 times per month	81 (27.6)	42 (51.9)	39 (48.1)	
Once or more per week	25 (8.5)	14 (56.0)	11 (44.0)	
Frequency of eating hot dogs				
None/Less than once per month	242 (82.3)	117 (48.3)	125 (51.7)	0.746
1–2 times per month	33 (11.2)	14 (42.4)	19 (57.6)	
Once or more per week	19 (6.5)	10 (48.0)	9 (47.4)	
Frequency of eating hamburgers				
None/Less than once per month	102 (34.7)	47 (46.1)	55 (53.9)	0.890
1–2 times per month	107 (36.4)	52 (48.6)	55 (51.4)	
Once or more per week	85 (28.9)	42 (49.4)	43 (50.6)	
Frequency of eating chicken				
None/Less than once per month	13 (4.4)	7 (53.8)	6 (46.2)	0.071
1–2 times per month	48 (16.3)	30 (62.5)	18 (37.5)	
Once or more per week	233 (79.3)	104 (44.6)	129 (55.4)	
Frequency of eating fish				
None/Less than once per month	21 (7.1)	9 (42.9)	12 (57.1)	0.625
1–2 times per month	81 (27.6)	36 (44.4)	45 (55.6)	
Once or more per week	192 (65.3)	96 (50.0)	96 (50.0)	
Frequency of eating green vegetables				
None/Less than once per month	12 (4.1)	6 (50.0)	6 (50.0)	0.801
1–2 times per month	54 (18.4)	28 (51.9)	26 (48.1)	
Once or more per week	228 (77.6)	107 (46.9)	121 (53.1)	
Frequency of eating tuberous vegetables				
None/Less than once per month	61 (20.7)	28 (45.9)	33 (54.1)	0.210
1–2 times per month	81 (27.6)	33 (40.7)	48 (59.3)	
Once or more per week	152 (51.7)	80 (52.6)	72 (47.4)	
Frequency of eating grains				
None/Less than once per month	28 (9.5)	16 (57.1)	12 (42.9)	0.543
1–2 times per month	95 (32.3)	43 (45.3)	52 (54.7)	
Once or more per week	171 (58.2)	82 (48.0)	89 (52.0)	
Consumption of delivery foods or food from outside the house				
Never	79 (26.9)	43 (54.4)	36 (45.6)	0.345
Once per week	164 (55.8)	73 (44.5)	91 (55.5)	
Twice or more per week	51 (17.3)	25 (49.0)	26 (51.0)	

Bolded data are significant

**Table 5:** Percent distribution of history of digestive diseases gastric neoplasms among participants

	Overall *n* = 294	*H. pylori* (−) *n* = 140	*H. pylori* (+) *n* = 154	*p*-value
Gastroenteritis				
No	260 (88.4)	129 (49.6)	131 (50.4)	0.116
Yes	34 (11.6)	12 (35.3)	22 (64.7)	
Peptic Ulcer				
No	228 (77.6)	126 (55.3)	102 (44.7)	**< 0.001**
Yes	66 (22.4)	15 (22.7)	51 (77.3)	
Esophagitis				
No	278 (94.6)	130 (46.8)	148 (53.2)	0.087
Yes	16 (5.4)	11 (68.8)	5 (31.3)	
Hepatitis C				
No	283 (96.3)	132 (46.6)	151 (53.4)	**0.022**
Yes	11 (3.7)	9 (81.8)	2 (18.2)	
Adenocarcinoma				
No	267 (90.8)	135 (50.6)	132 (49.4)	**0.005**
Yes	27 (9.2)	6 (22.2)	21 (77.8)	
Gastric MALT lymphoma				
No	281 (95.6)	136 (48.4)	145 (51.6)	0.483
Yes	13 (4.4)	5 (38.5)	8 (61.5)	
Stage of Gastric Cancer				
Early	20 (6.8)	7 (35.0)	13 (65.0)	0.456*
Advanced	16 (5.4)	3 (18.8)	13 (81.3)	
Lymph node metastasis				
No	24 (8.2)	8 (33.3)	16 (66.7)	0.711*
Yes	13 (4.4)	3 (23.1)	10 (76.9)	

*p-value of Fischer Exact test

Bolded data are significant

**Table 6:** Unadjusted and adjusted odds ratios of *H. pylori* infection status with various factors

	Unadjusted OR (95% CI)	Adjusted OR (95% CI)
Age (mean ± SD, years)	1.00 (0.98–1.01)	–
Sex		
Males	1	–
Females	0.80 (0.50–1.29)	–
Marital status		
Non married	1	–
Married	0.93 (0.57–1.53)	–
Education		
Below high school	1	1
High school	1.57 (0.77–3.17)	2.17 (0.84–5.60)
University and higher	1.49 (0.81–2.76)	2.74 **(1.17–6.44)**
Income		
< 660 USD	1	–
≥ 660 USD	0.80 (0.50–1.27)	–
Glycemia		
Normal (≤1.2 g/L)	1	1
Above normal (> 1.2 g/L)	0.26 **(0.08–0.83)**	0.18 **(0.03–0.89)**
Vitamin D level		
Normal (≥20 nanog/L)	1	1
Below normal (< 20 nanog/L)	24.57 **(10.78–56.03)**	29.14 **(11.77–72.13)**
Frequency of drinking milk		
None/Less than once per month	1	–
1–2 times per month	2.23 **(1.21–4.10)**	–
Once or more per week	1.40 (0.79–2.49)	–
Peptic Ulcer		
No	1	1
Yes	4.20 **(2.23–7.90)**	3.80 **(1.80–8.01)**
Hepatitis C		
No	1	1
Yes	0.19 **(0.04–0.92)**	0.18 (0.02–1.43)
Adenocarcinoma		
No	1	1
Yes	3.58 **(1.40–9.15)**	3.99 **(1.35–11.83)**

Bolded data are significant

## Discussion

Prevalence of *H. pylori* infection in this study was found to be 52.4%. The risk of having the infection was significantly higher among subjects with an educational level of university or higher, with normal glycemic levels, and those with vitamin D levels below normal, after adjusting for other confounders. No association between *H. pylori* status and dietary habits was detected. Findings of this study might help clinicians make better informed decisions on treatment options based on their patients’ dietary and lifestyle habits.

Our estimate of *H. pylori* infection is comparable to the prevalence of 52% reported among the general Lebanese adult population by Naja et al. [28]. This rate is lower than that found in other countries of the MENA region including Egypt, Libya, Saudi Arabia, Iran, Oman, United Arab Emirates, and Turkey where the prevalence of *H. pylori* ranged between 70% and 94% [10, 15, 29]. The only exception was a study conducted in Gaza, Palestine where *H. pylori* prevalence was found to be 48.3% [30]. Compared to other studies among symptomatic patients with dyspepsia or other GI symptoms conducted in this region, a review article by Khedmat et al [29] showed that studies in all countries had a higher prevalence ranging from around 70% up to 100%, except for one conducted in Jordan on 250 patients undergoing a biopsy on a specimen of the gastric antrum, reporting a prevalence of 44%. Other developing countries in Asia had prevalence rates similar to those reported in this study [10]. On the other hand, prevalence of *H. pylori* infection in Lebanon is still higher than the rates reported in developed countries including Canada, USA, Australia and Western European countries with rates that range from around 11% in Sweden to 48.8% among older adults in Germany [10].

Subjects with university degree or higher had almost three times increased risk for *H. pylori* infection (OR = 2.74; CI = 1.17–6.44). The literature is inconsistent on the association between education level and H pylori, with some studies showing no association while others reporting a higher risk for *H. pylori* among subjects with lower education level. Naja et al. in a cross-sectional study conducted in Lebanon on 308 participants reported no association between education level and *H. pylori* [28]. In addition, a prospective study conducted on 516 asymptomatic subjects showed no association between *H. pylori* infection and educational level in Pakistan [31]. Similarly, Fani et al [32] and Aguemon et al. [33] reported no relationship between *H. pylori* infection and education. In contrast, a cross-sectional study on 19,272 subjects aged 16 years or older in South Korea, reported that those with high education level and high income were less likely to be *H. pylori* seropositive [34]. Also, prevalence of *H. pylori* infection in Vietnamese migrant women was lower (55.7%) than that of national Korean females (71.4%). Migrant workers in large cities of Northern China were also tested for *H. pylori* infection and had a low rate of infection (41.5%). Indigenous populations in Northwestern Ontario in Canada, had a lower prevalence than expected (37.9%) [35] . On the other hand, this result might be due to variations in study design, ethnicities of the sample, the designated tests used to estimate prevalence, symptomatic versus cross-sectional volunteer patients, or use of suppressive medications among studies. More research is needed to investigate whether this result is due to chance or to other unknown confounding factors.

High glycemia was negatively associated with *H. pylori* risk (OR = 0.18; 95% CI = 0.03–0.89). The relationship between diabetes mellitus and *H. pylori* infection is not well established in the literature. A meta-analysis of 11 studies including 513 patients with diabetes mellitus has shown that *H. pylori* negative status was significantly associated with lower glycosylated hemoglobin (HbA1c) levels (WMD = 0.43, 95%CI: 0.07–0.79), and a meta-analysis of 6 studies including 325 type 2 diabetic patients has shown the infection to be associated with higher fasting plasma glucose (WMD = 1.20, 95% CI: 0.17–2.23) [36]. However, eradication of *H. pylori* has not shown to improve HbA1c or glucose levels after a period of 3 months or 6 months [36–38]. On the other hand, a study conducted in Lebanon to examine the relationship between metabolic syndrome and insulin resistance with *H. pylori* found that hyperglycemia was not significantly associated with the infection [28]. In addition, Jafarzadeh et al. reported *H. pylori* seropositivity rates that were similar between participants with type 2 diabetes (76%) and healthy subjects (75%) in Rafsanjan, Iran [39]. Results from the Netherlands were also similar [40]. Interestingly, the eradication of *H. pylori* in a case-control study showed a significant increase in the incidence of obesity, hypercholesterolemia and hypertriglyceridemia after 1 year of the treatment [41]. In a review of the evidence regarding the association between *H. pylori* and extragastric manifestations, Suzuki et al. [42] concluded that in the case of diabetes mellitus, the clinical consequences of *H. pylori* infection in terms of metabolic control seems to be low. So this explanation might also fit pre-diabetes. Moreover, Lutsey et al. [43], using data from the Multiethnic Study of Atherosclerosis reported a lower rate of *H. pylori* infection in patients with diabetes, consistent with our results. It remains uncertain how *H. pylori* serostatus affects the pathogenic process leading to metabolic syndrome. This surprising finding might be attributed to the fact that persons with insulin resistance (high glycemia) might be asked to modify their diet upon their diagnosis, and so begin to eat less fatty food items and increase their fruit and vegetable consumption which promotes probiotic populations versus *H. pylori* infection.

Participants with below normal levels of vitamin D were more likely to be infected with *H. pylori* than those having normal vitamin D levels. Few studies have investigated the role of vitamin D in preventing *H. pylori* infection. A case-control study on women aged 70 to 99 years has shown that long-term supplementation of 1 alpha-hydroxyvitamin D-3 as part of osteoporosis treatment significantly inhibited the development of the infection [44]. A cross-sectional study conducted in Iran on patients with end stage renal failure who are on hemodialysis showed an association between serum 25-OH vitamin D and serum *H. pylori* specific IgG antibody titers, suggesting that vitamin D increases the immune response [45]. In fact, a recent article has demonstrated that a decomposition product of vitamin D3 has an antibacterial effect against *H. pylori* bacteria specifically [46]. This area is worth more investigation as vitamin D supplementation might be effective in treatment and prevention of H pylori infection. In fact, since long time ago, vitamin D deficiency has been suggested to increase the risk for infections, as it was observed that children with rickets were more prone to respiratory infections. This is explained by the modulating role of this vitamin in the immune response, as more recent studies have also shown that the incidence of different infectious diseases, including influenza, respiratory infection and septic shock might be due seasonal variations in vitamin D levels as exposure to solar ultraviolet-B doses is lower during winter [47].

The link between *H. pylori* and a wide range of upper digestive diseases including peptic ulcer and gastric cancers has been well established in the literature. Indeed, peptic ulcer and adenocarcinoma were significantly associated with *H. pylori* infection in this study. Furthermore, a meta-analysis of 52 trials has shown that eradication of *H. pylori* is effective in treating duodenal and gastric ulcers and decreasing their recurrence [48]. *H. pylori* has been found to increase two times the risk of developing gastric adenocarcinoma according to a meta-analysis that included 42 studies [49]. This is consistent with more recent research showing that patients with *H. pylori*-positive non-atrophic gastritis are at around 10 fold higher risk to develop peptic ulcer and twice higher risk for gastric cancer compared to healthy individuals [50].

None of the food items studied was associated with *H. pylori* infection. The relationship between different food items and *H. pylori* infection remains inconclusive. Consistent with our findings, a recent cross-sectional study conducted in Oman on 100 patients attending Sultan Qaboos University Hospital showed no correlation with the intake of any of the studied food items with the exception of soft drinks [21]. However, meat and fast food consumption were significantly associated with *H. pylori* infection in other studies conducted in Iran and India [11, 22]. Also, while some studies have shown that fruits and vegetables intake decreases the risk for *H. pylori* infection [17, 19, 22], others have not [20]. More randomized controlled trials should be conducted to explore further the effect of different food components on *H. pylori* eradication. Such research would identify healthier alternatives for treating *H. pylori* colonization than pharmacological therapy that has side effects and leads to antibiotic resistance [51]. On the other hand, Xia et al. argue that it is important to study dietary patterns and not food items in isolation, since nutrients do not only act independently but may also interact together ^[52]^ since *H. pylori* was positively association with a diet rich in carbohydrates and sweets, and negatively associated with a diet high in protein and cholesterol, while no association was found between *H. pylori* and any food items or groups studied in isolation in their cross sectional study.

Some limitations of the study should be considered when interpreting the results. A convenience sampling method was used to select participants, thereby limiting the ability to generalize results to the target population. Potential misclassification bias of the main outcome although minimal is possible, Serological testing, could be improved further by using stains having higher sensitivity and specificity than H&E stain such as Giemsa stain, Warthin-Starry silver stain, Genta stain, and immunohistochemical (IHC) (69–93% and 87–90% respectively, versus 90–100%) [52, 53]. Moreover, the FFQ administered presents some limitations. Food intake was self-reported with no means of verification, leading to a potential information bias. In addition, intake frequency of specific food items was assessed without specifying quantities or portion sizes. However, it is believed that the variation of portion sizes between different participants is smaller than that of frequency of intake, and thus would have limited impact on the results [54]. Small sample sizes in some of the independent variables might have led to inflated risk estimates and significant results which may be spurious. Finally, the cross-sectional design prevents the inference of inferring causality. This study has several strengths. This is the first study in Lebanon and one of the few in the region to analyze the association between *H. pylori* infection and dietary habits while adjusting for potential confounders. Biopsy has higher specificity than serological testing for assessing infection presence, thereby minimizing misclassification bias [55]. Anthropometric measurements, blood tests results and certain medical conditions were abstracted from patients’ charts, eliminating self-report bias. Moreover, an FFQ previously validated by Yassibas et al. [26] was employed to assess general dietary intake; FFQ is considered to be the most appropriate dietary tool for studying the relationship between diet and disease. Finally, as less than 2% refused to participate in the study, non-response bias was negligible.

## Conclusion

*H. pylori* infection is a major public health issue affecting more than half of world population and leading to a range of gastro-intestinal problems. This study is the first in Lebanon and one of the few in the MENA region to examine the dietary correlates of *H. pylori* infection. University education was a risk factor for *H. pylori*. None of the food items or dietary habits was associated with *H. pylori* infection. However, adequate blood level of vitamin D was found to protect against it. Conversely, participants with hyperglycemia were at decreased risk of *H. pylori*, an uncommon association that needs to be investigated further. It is essential to study the treatment potential of dietary substances that appear to have a protective effect against *H. pylori*. This would present a solution with lower cost, higher availability and fewer side effects than medications. Our results, in addition to findings from other studies, suggest that vitamin D supplementation might be one healthy alternative, but more longitudinal studies are needed to confirm its effectiveness. In addition, cohort studies examining the link between *H. pylori* and dietary patterns rather than isolated food items are needed to take into account nutrients interaction. Such studies would help clinicians make better informed decisions based on their patients’ dietary and lifestyle habits. It would also allow designing health education interventions that promote general recommendations on healthy eating patterns rather than specific food components intake.

## Abbreviations

FFQ: Food frequency questionnaire
*H. pylori*: *Helicobacter pylori*
MENA: Middle East and North Africa
NSAID: Nonsteroidal anti-inflammatory drug
PPI: Proton pump inhibitors
